# Enter into society: Digitalized livelihoods and prosumer labor for people with disabilities in China

**DOI:** 10.3389/fpsyg.2023.1103199

**Published:** 2023-03-17

**Authors:** Yaxiong Bao, Xinyu Hu, Ruijie Zhang, Xiaochuan Shi

**Affiliations:** ^1^School of Journalism and Culture Communication, Zhongnan University of Economics and Law, Wuhan, Hubei, China; ^2^School of Cyber Science and Engineering, Wuhan University, Wuhan, Hubei, China

**Keywords:** digital labor, prosumer labor, people with disabilities, digital economy, social exclusion

## Abstract

**Background:**

The current debates on “digital labor” focus on a gorgeous and comprehensive experiential description and theoretical exposition but do not provide a thorough examination of the unique context and social structure. In China, the development of internet is closely tied to politics, the Chinese Government uses internet as a tool of social governance. More importantly, aside from desire-based communications produced by corporate ideology, the Chinese people’s passion for the Internet also comes from individual survival, especially the middle and lower class of information represented by the disabled people. This means that analysis of the digital labor among people with disabilities in China must be done from a variety of angles, including politics, society and culture.

**Methods:**

This study combines life-history interviews and field research methods to explore the value and significance of digitalized livelihoods and free prosumer labor for people with disabilities in China through self-narration. Since 2020, researchers have been volunteering at two social organizations serving people with physical disabilities in Wuhan city, Hubei Province. We participated in 26 assistance activities for disabled groups which included three 14-day training camps, and interviewed 40 people with physical disabilities.

**Results:**

This study found that although the digitalized livelihoods practice of people with disabilities is still “precarious labor” in nature, whose self-expression in the cyberspace is easy to fall into the shackles of capital flow logic. However, digital labor practice provides them with the opportunity to “sit at home, enter the community and society,” also enables them to “live independently.” More importantly, this opportunity and possibility enable people with disabilities to experience a sense of value and self-esteem as “competent people.” Therefore, in the realistic environment of structural obstacles in the social life of the people with disabilities in China, the possibility of “inclusiveness” brought by digital labor is the core value brought by digital society.

## 1. Introduction

The formal introduction of the Internet in 1994 marks the beginning of China’s 28-year history of digital development. One after another, the Internet, computers, cellphones, artificial intelligence (AI), and other information and communication technologies (ICTs) have emerged as fundamental tools for social production (Zhang, 2021), having a significant impact on the general public with varying levels of wealth. Some scholars refer to the manner that people live after the extensive integration of ICTs into economic and social life as “medialized existence” (Sun, 2020). What does this way of life entail for people with disabilities who have been excluded from social structure, information access, and even social production?

Approximately 85,000,000 people in China were reported to have disabilities in 2010, according to the China Disabled Persons’ Federation (CDPF) (CDPF, 2012). That means on average, one out of every 16 people has a disability. The vulnerable group of people with disabilities has been referred to as “the sleeping giant among people” due to their vast numbers and relative isolation from mainstream society (Zola, 1993). Therefore, it is essential to encourage their growth, equality, and engagement in society. As the Internet has grown in popularity and the digital economy has developed quickly, more academics have become interested in debating the proper place of digital technology in modern society and have developed two opposing viewpoints. Those technophiles have the view that digital technology possesses the potential for empowerment, which can rid society of its hierarchical structure and create a world characterized by “consultative democracy, sharing, and cooperation.” The public is becoming “dialogists” and “participants” in this universe, getting rid of the function of “passive receivers.” In contrast, the majority of researchers who use political economics of communications as their research paradigm rely on the theoretical framework of “exploitation,” emphasizing the need to expose and denounce how capital exploits Internet users in the age of new technology through more cunning and subtle means. This has resulted in the development of pertinent notions such as the precariat (Standing, 2011), free labor (Terranova, 2004), playbour (Kücklich, 2005), immaterial labor (Hardt and Negri, 2005), platform labor (Huws et al., 2018), and the digital working class (Qiu, 2018). The differing perspectives on Internet workers serve as a reminder of the significance of analyzing “digital labor” in light of the living conditions of the general public from various cultural and social classes.

The current debates on “digital laborers” focus on a gorgeous and comprehensive experiential description and theoretical exposition but do not provide a thorough examination of the unique context and social structure. Political ecology in China is closely impacted by the development of Internet technology. Even the Chinese government now uses the Internet as a key tool for social administration. The “Guiding Opinions on Promoting ‘Internet Plus’ Initiative’“document, which recognized “Internet Plus” as a national economic strategy, was published in 2015 by the State Council of the People’s Republic of China. According to the statement, it is essential to “accelerate the deep integration of the Internet and many fields to establish a new development form for economic and social development.” The employment of the handicapped can be better ensured by these policies. The “Program for Accelerating the Handicapped to Live a Better-Off Life during the “13th Five-year Planning” “was released by the State Council of the People’s Republic of China in 2016, and it makes it abundantly apparent that the handicapped are encouraged to launch their own businesses and look for work using “Internet Plus.” Thus, “government purchase+e-commerce service” is a novel form of employment assistance model that has successfully encouraged handicapped people to look for self-employment online. This appears to offer convincing evidence for the “promise of technology,” or the potential of ICTs to enhance the quality of life for the handicapped and social acceptance of them (Foley and Ferri, 2012). Aside from desire-based communications produced by corporate ideology, the Chinese people’s passion for the Internet also comes from survival-based communications. People utilize the Internet for survival, employment, and communication purposes. The desire for survival is more apparent to the middle and lower classes of the information world represented by the handicapped, which can have a direct impact on how they see their own worth, confidence, and self-esteem (Lin et al., 2019). This means that analysis of the digital labor among the handicapped in China must be done from a variety of angles, including politics, society, and culture. By doing this, a thorough interpretation of the particular function performed by “digital labor” from all angles may be ensured. In this paper, we study the digital life of people with defective limbs after their limbs are amputated. We also incorporate social macroscopic policies and the unique characteristics of the handicapped into our research. We carefully examine the worth and significance of the handicapped’s digital labor as well as the impact of digital labor on each person’s psychology using semi-structural interviews and observation. In the meantime, the essence of the contrasts between the handicapped’s digital labor and earlier types of labor are determined.

## 2. Literature review

### 2.1. Research on digital labor

In the 1980s, the arrival of the Internet brought audience research into a novel research context, and the “active audience view” as well as “audience power view” were pushed to a new climax in the Internet era. Decentralization, empowerment, and participation in decision-making have become the buzzwords of technical optimists. Alvin Toffler creatively put forward the concept of prosumer rather than audience, and developed the audience from a single consumer in the media industry to the subject of production and consumption. Afterwards, Alex Bruns further developed this concept into produser, emphasizing the dual attributes of netizens’ production and use of information, and affirming the subjectivity and initiative of netizens. Nevertheless, in the eyes of numerous researchers of communication political economy, the discourses of “users,” “participation” and “sharing” are precisely the persuasive business ideology created by the alliance of Internet communication technology and commercial capital.

On this basis, researchers began to explore the game of multiple forces on the commercial social media platform (Fuchs, 2014), paying attention to the exploitation of free labor of netizens by capitalist production methods (Terranova, 2004) and the necessity fordigital workers to unite for class struggle (Sandoval et al., 2014). They adopted the concepts of free labor and unwaged intangible labor to highlight the dual attributes of digital labor, namely, autonomy and gratuitousness. Generally speaking, there are two primary directions in the research of digital labor currently. One is the research on digital labor with digital practitioners as its main research object, and the other is the research with the prosumer digital labor of non-employed forms of digital users as the research object. The former continues Marx’s early understanding of creative labor in political economy. It advocates a focus on the labor control process of professional labor in digital industry to criticize the exploitation of the labor value of professional labor induced by the accumulation of capital in new fields. For example, Ross (2004) suggested that the Internet industry, stemming from freedom, cooperation, and democracy, superficially granted the Internet engineers a high degree of freedom, autonomy, and equality in their work experience, whereas behind it were dehumanizing working conditions such as intense overtime, strong pressure, stricter self-management, and blurred boundaries between work and leisure. Contrary to “knowledge-based workers” such as well-paid programmers and UI designers, a great deal of researchers have also explored platform labor such as Uber drivers (Rosenblat and Stark, 2016) and delivery riders (Chen, 2020), arguing that in the world of platform labor, inequality is a feature rather than a bug, and platform labor remains thoroughly embedded in the world created by capitalist value forms (Van Doorn, 2017). In addition, several domestic scholars also pay attention to the existence of a large number of network water forces employed by commercial marketing companies in China, which are considered to be the institutional product of contemporary China digital society. The digital resources such as netizens’ fragmentation time, credit rating and identity information have been transformed into digital capital that can be clearly priced and traded. On the whole, these studies emphasize the “illegality of coercion,” injustice (Fuchs, 2014) and “manufacturing consent” (Burawoy, 1982) in the process of digital workers’ labor, reckoning that precarious labor (Standing, 2011) is the essential feature of various types of labor such as wage labor and platform labor in the context of digital capitalism. The latter originated from the theory of audience commodity proposed by Smythe (1977) focusing on productive consumption labor, particularly on netizens. Smythe’s (1977) audience commodity theory argues that the media gain profit by selling “audience’s attention” as a commodity to advertisers. In this case, the behavior of the audience watching advertisements constitutes “audience labor.” With the advent of the Internet era, Smythe’s viewpoints gained a group of supporters in the field of digital labor research, then concepts such as User-Generated Content (UGC) and the unity of prosumers and producers flooded in accordingly. Related studies focus on the forms of digital labor including data production, affective labor, and crowdsourcing labor by netizens, and emphasizes that the digital users are prone to be exploited when they reproduce their labor in non-wage time. For example, Wu (2022) focused on UGC on the Internet platform, revealing that “UGC under the business ideology of “sharing” and “participation” creates a false power imagination for netizens, and its essence is the enhancement of capital profit-seeking. Regarding the usage data of netizens, Chandler and Fuchs (2019) proposed that platform companies would exploit and oppress digital users by monitoring their behaviors and social networks and screening a large amount of data beneficial to advertisers. De Peuter and Dyer-Withefordk (2005) and Jin (2017) took the “playbour” of video games as the research object, pointing out that the players’ enthusiasm and creativity for games would be exploited by game companies in game development and post-maintenance, thus forming the exploitation of players as free labor. However, some studies emphasize the “positive emotion”of users in the process of content production from the perspective of “affective labor” (Hardt, 1999). For example, McCosker and Darcy (2013) targeted to cancer blog, a kind of UGC, which, expands the theory and debates around the nature of immaterial and affective labor, and highlights the value of cancer blogging as an individual, in the form of identity and affect management, network-enabling in generating online spaces for shared experience and support, and social value of compensation in the form of non-institutional management of serious diseases.

The aforementioned research provides two dimensions for exploring the digital labor of people with disabilities. The labor for monetary gains on the Internet platforms by the disabled group as digital practitioners and digital labor of production and consumption type made by ordinary users during the process of using the Internet. Therefore, the paper will analyze how the digital labor of people with disabilities influences their daily life in terms of their “digitalized livelihoods” and “prosumer digital labor.” For the purpose of avoiding falling into a single framework of “exploitation” or “empowerment,” this study introduces a “subjectivity” perspective to deeply understand the needs, anxieties, and aspirations of persons with disabilities from their own narratives, to discuss how they perceive their digital labor, and assess the gains and losses of it.

### 2.2. Digital media practices for disabled groups

The discovery of the phenomenon of the digital divide and its emergence as an academic concept in the 1990s had a significant influence on domestic and international research on digital media in underserved communities. Digital disability research responds to core issues including the digital divide, digital inclusion, as well as technological empowerment, which gives rise to popular interdisciplinary concepts, namely, universal design and assistive technology (Zhang, 2021). These studies focus on the “new divide” and “new opportunities” for the social integration of disabled groups brought about by Internet technology. For instance, Kent and Ellis (2015) pointed out that the increasingly complex and overlapping digital communication platforms made it challenging for individuals with disabilities to obtain disaster information, and believed that complex information acquisition could endanger the lives of individuals with disabilities at the time of disaster. In contrast, numerous disabled researchers emphasize Internet-enabled equality of access to information, the possibility of a remote “home office” and the opportunity to hide their physical characteristics and overcome prejudice. They believe that the Internet is a utopia for people with disabilities who are absent from traditional society, as it can improve the social communication of disabled groups and compensate for the barriers caused by education, class, and environment that prevent them from in society. Boellstorff (2019) also noticed that the digital labor experience of disabled people challenged the dualism of “ability” and “disability” and reshaped the functional relationship among self, work, and value. As an essential platform for disabled groups to interact, communicate and initiate rights protection actions, Lee and Cho (2019) found that social media can facilitate the formation and maintenance of friendships between disabled groups and improve their mental health. Moreover, disabled people not only try to explain their loneliness, disability discrimination, and cultural devaluation in online communities but also begin to pay close attention to social injustice and advocate their rights through social media, which indicates the potential of social media to reshape disability politics and develop new forms of disability activism. Additionally, the rapid development of mobile networks and the introduction of disabled applications make mobile media including smartphones and tablets become new types of assistive devices for the disabled. Due to its “normal” appearance, mobile media would not bring disability discrimination like assistive devices including wheelchairs, and it is a more “dignified” assistive device. Furthermore, since the reform and opening in 1978, the Chinese party-state employs neoliberal discourse for the purpose to encourage individualized citizens ‘to be self-responsible, self-enterprising, as well as self-governing subjects and established cultural conventions and legal obligations including “labor is the only source of creating value” and “labor is the glorious duty of every citizen who has the ability to work.”

According to the aforementioned research, the digital life of the disabled population is impacted by both embodied damage and social disability factors, different usage scenarios and technological adaptability will lead to completely different results. As Macdonald and Clayton (2013) stated, this paper emphasizes the embodied experience of people with disabilities using digital technologies and focuses on the influence of individual differences in digital practices resulting from a physical condition, generation, gender, geography, education, family, as well as other factors.

### 2.3. Digital media practices and individual psychology

The paper is expected to sort out the existing research on digital media practices and individual psychology according to two aspects of digital labor research. In terms of digital practitioners, Neff (2012) reckoned that the rapid development of the IT industry and high technology brought about high-pressure and high-intensity labor control, making the knowledge laborers in Internet companies anxious about “eating their youth.” Mosco (2018) argued that, with a focus on the working environment of Internet content managers, these digital laborers have been left with severe post-traumatic stress disorder (PTSD) due to long-term exposure to pornographic content. In terms of the prosumer labor of Internet users, existing research has found that social media interactions can reduce users’ loneliness (Burke et al., 2010) and increase self-efficacy and life satisfaction (Valenzuela et al., 2009) by lowering barriers and thresholds for maintaining strong or weak relationships (Ellison et al., 2007) and increasing bridging social capital and connected social capital. Users are more likely to make new social connections and receive more positive feedback, such as “likes”(Yang and Brown, 2016) than those who do not use social media, along with the improvement in well-being (Valkenburg et al., 2006). Of course, it has also received skepticism from researchers with a cyber-pessimistic attitude, who argue that online activities can negatively affect users’ psychological well-being (Kraut et al., 1998) by increasing loneliness through reduced social engagement. These studies show the completely different effects of digital media on individual psychology and remind us to attach more importance to the specificity of digital media use experiences of different groups in adaptation to realistic situations. However, most of the above studies are mainly quantitative and lack vivid and rich individual life stories. Therefore, this paper is expected to analyze the impact of digital practices on the psychology of people with disabilities in China through their self-narratives and life stories on the basis of considering the structural dilemma they face.

## 3. Research perspective, research methods, and data collection

Taking the life course perspective of the sociology of disability as an analytical lens, this paper explores the value and meaning of digital labor for disabled people as well as its impact on their psychology. The life course perspective not only emphasizes the integration of individual life experiences with broader social institutional arrangements, but also focuses on how social institutions and culture further influence the life experiences of disabled groups (Priestley, 2003).

The paper focuses on people with physical disabilities in Wuhan, Hubei, China, and collects data using in-depth interviews and observation methods. Since 2020, researchers have been volunteering during vacation time at two social organizations serving people with physical disabilities in Wuhan, which often hold occasional activities to help disabled people. During the service, researchers learned that almost every disabled person uses social media such as “Tik Tok” and Internet forums. In addition, some of them even make use of the Internet to earn a living. This phenomenon prompts the researchers’ interests in studying the digital labor of disabled people. After submitting the research design to the Academic Committee of the School of Journalism and Cultural Communication of Zhongnan University of Economics and Law and gaining the approval of the ethical review, researchers began to observe and learn about how disabled people use Internet.

The ways of observation method adopted by this research are mainly embodied in the following two aspects: First, researchers observed and recorded the behavior of disabled people using the Internet when participating in disability assistance activities. Apart from the brief daily assistance services, researchers participated in a total of 3 “spinal cord injury training camps” activities from December 2020 to June 2022, organized by the Wuhan Disabled Persons’ Federation and provided by social organizations for the disabled, with each lasting 14 days, and involving as many as 20 trainers and participants (all of them were physically disabilities). During the camps, researchers lived with disabled people as volunteer and observed up close to keep track of their daily use of Internet. On the other hand, during the assistance service, researchers met and followed many disabled people through social media, analyze their self-produced content on social media.

Based on the retrospective accounts of the interviewees, the in-depth interviews understand the key events experienced by people with physical disabilities during the development of the Internet in China, and thus portray the changes in their digital labor in response to changes in national policies, the socio-economic development, the media environment, and individual circumstances. It is considered that narrative is a medium to express and reflect on personal life experiences (Plummer, 1983). This approach is often applied to qualitative research. Compared to pre-determined questionnaires, narrative inquiry provides a deeper understanding of the meaning of ICTs in the lives of disabled people. During the research period, researchers interviewed a total of 40 people with physical disabilities. Applying the replication logic in case study for respondent screening, 16 respondents were recruited in this paper. Please see Table 1 for demographic data. Replication means the repeated appearance of the same cognition or phenomenon among different respondents. During the process of paper writing, for an opinion or behavior repeatedly mentioned by different people with disabilities, only one or two typical stories of people with disabilities were chosen as evidence. For example, there were 12 respondents mentioning that the economic development of platforms brings life convenience. However, this paper only illustrates the significance that the disabled use the Internet platforms with the stories of YJ and FF.

**Table 1:** Sociodemographic profile of 16 interviewees.

Age categories	26–50 years
Causes of disability	12 respondents were physically disabled due to acquired diseases or injury, and 4 respondents were congenitally handicapped.
Gender Distribution	Ten men and six women
Level of disability	12 respondents were at the level of 1–2, and 4 respondents were at the level of 3-4(according to Chinese disability recognition standards, level 1 is the most severe and level 4 is the least severe)

We maintained standard ethical protocols to conduct this research. At the beginning of the interviews and observations, each participant was briefed on the content of the study, and informed consent was sought from them. The identities of the participants were kept confidential, all the names of the interviewees are replaced by English letters in this paper. We assured that the information provided by the participants would only be used for academic research.

## 4. Digitalized livelihoods for people with disabilities

Unlike the ordinary people’s life course of “receiving education–hunting for job–employment–retirement,” employment for people with disabilities has always been unstable and inadequate. For the common man, becoming a digital practitioner makes it accessible to free working hours, good pay, and a better life, but for people with disabilities, it is simply a way to keep themselves and their families alive. Therefore, this paper uses the term “livelihood” rather than “work” or “labor” to refer to the “form of livelihood” that people with disabilities make a living through the Internet. That is because the term, livelihood, implies a means of earning basic living necessities including food, clothing, shelter, and transportation.

### 4.1. The reality of employment difficulties due to social exclusion and physical functioning

There are three main types of disability employment models in China’s mainland: collective employment, dispersed employment on a proportional basis, and individual self-employment. After the founding of the People’s Republic of China (PRC) in 1949, the Chinese government set up state-run welfare factories and government departments to embrace people with disabilities specifically, and to give access to the same possibilities of labor rights, pay and career promotion as non-disabled people. However, such opportunities for steady jobs are only available to a minority of well-educated people with disabilities domiciled in urban areas. HWZ, a person with physical disability the author met in field research, joined the Hubei Provincial Disabled Persons’ Federation because of his master’s degree and experience in overseas studies. The wave of marketization, beginning in the 1990s, has led to the closure of most state-run welfare factories and the reduction of government job openings, leaving urban workers with disabilities with fewer opportunities for stable jobs. In this context, the Chinese government has introduced policies to boost the employment rate for people with disabilities, such as pro-rata dispersed employment. Relevant policies require employers to employ people with disabilities at a rate of not less than 1.5% of the total number of employees in the unit, and those who do not comply with the regulations are subject to a certain amount of employment security fund for people with disabilities. However, the jobs derived from these employment policies do not meet the needs of the vast number of people with disabilities in Chinese society. In accordance with 2020 Statistical Bulletin on the Development of the Work for Persons with Disabilities released by the China Disabled Persons’ Federation, the employment rate of Chinese disabled people was only 43.24% in 2020. From the interviewees’ narratives in the field survey, social exclusion and physical functioning limitations and the psychological trauma caused by it are the significant difficulties in their employment.

The first is employment exclusion. In the traditional occupational system, people with disabilities are often regarded as handicapped and disabled persons, many employers have a stereotype that they are less sociable, hard to integrate, and with low-level work completion. Moreover, in the workplace culture from the ability-oriented perspective, people with disabilities who are not able to work overtime regularly or even sit in the office for long periods are seen as a “nuisance.” Therefore, they do not take into consideration hiring people with disabilities given the options available. NY, one person with physical disabilities with an human resource management (HRM) undergraduate major, described his job-hunting experience as follows:

*“I have submitted many resumes, and once I was called for an interview by a cultural company. When I told the interviewer over the phone that I was disabled, he replied directly that he was sorry and did not expect me to come to the interview anymore. And once, I passed an interview of a non-profit organization. The first day I went here, I found my position at the innermost part of the office, difficult to reach in a wheelchair. Then the kind leader asked my colleague to change the position with me. But I went to the washroom, and it was too narrow for my wheelchair, so I resigned after two days.”*

It is evident that hiring preferences of recruiters and social exclusion from the accessible environment play an important role in the fair access to the job market for disabilities.

The second is limitations of physical functioning. In the exploration of social interaction among people with disabilities, some researchers reckon the fact that social networks are shrinking not only because modern means of production do not offer reasonable, convenient, and suitable posts for people with disabilities, but also because of the physically challenged body itself (Corker and Shakespeare, 2002). For people with severe disabilities, in particular, the time manage their bodies significantly crowds out the time and effort that could be devoted to social participation.

*“After paraplegia I was incontinent, and I was slow to get dressed, wash my face and do other activities. What an able-bodied people can do in 5 minutes, may take us 50 minutes. I usually have to prepare diapers and wear adult diapers when I go out, I don't dare to drink water, because incontinence smells bad, thus easily getting disgusted by others. In addition, it is easy to hurt my buttocks after sitting in a wheelchair for a long time, leading to pressure sores. But I can't find a ready place to lie down for a while when I'm outside. So I'm a bit scared when I think of going out”,* said XM, a physically challenged person with a traumatic spinal cord injury resulting in paraplegia.

These private, trivial and inadequate body management activities wear them down, damage their self-esteem, and directly lead to their fear of going out and sense of inferiority, making them embarrassed to enter public areas. This kind of mentality makes them feel lonely and doubt whether they can work like common people. More importantly, in the context of socialist industrial production, the disabled have always been in a very low social class because they cannot involve in social production. In the meantime, the disabled are often described as useless and disabled. Such social discrimination has caused psychological trauma to the disabled group in the aspect of employment practices, and aggravated their trend to exclude themselves from the society, so as to avoid social harm caused by others.

*“The disabled are called cripples in the society, which means that we contribute nothing to the society. When we go out, strangers will stare and even laugh at us. Therefore, I dislike going out, let along working in a physical unit.”*

(YX with physical impairments)

According to these narrations, for the disabled, physical handicap and social discrimination deprive them of employment opportunities in the traditional society and cause more psychological barriers to their integration into the job market. Except for the shortage of employment opportunities and unfriendly employment environment in the real society, they need to eliminate physical and psychological obstacles to their employment realization.

### 4.2. Life opportunities from digital China

Before the rise of the digital economy, it was difficult for China’s people with disabilities to find work outside of the collective employment model. Many middle-aged people with disabilities said they were constantly shifting between part-time, entrepreneurial, and non-employment status, with grocery stores, shoe repair, and motorized tricycle rides as their primary means of earning a living. However, similar ways of making a living by taking passengers by electric vehicles were prohibited by Chinese law due to safety hazards. Many interviewees described their earlier lives with difficulty of finding a way to make a living and a serious lack of money. Even some of them had to rely on the subsistence allowance and financial subsidies from their families to make ends meet (On April 1, 2022, the minimum living standard for urban households in Wuhan was raised to 910 yuan per month).

The growth of Internet technology and the digital economy has developed innovative solutions for people with disabilities to make a living. Given that ICTs have removed some of the physical, psychological, and environmental barriers that people with disabilities face in the job market by changing the workspace and work style, many of people with disabilities regard internet-based livelihoods as the suitable way for them.

*“We can't move easily. So if we go to work outside, there are problems in eating and getting to the washroom, and our bodies can't take it. But if we earn money at home through the Internet, it's much more convenient, and we don't have to care about others' opinions.”*

(ZQ, a person with physical disabilities)

There is a vital vein in the life narrative of the interviewees about getting online, that is, “the history of my employment *via* the Internet.” Their course of online employment has witnessed changes along with the development of the Internet, and it can be said to the history of the development of the Internet in China.

Before social media such as Weibo, Zhihu, Douban and other C2C online retail platforms such as Taobao developed and grew in China, many people with disabilities were already finding ways to make a living through the internet. Forums for disabilities offered the most prominent opportunities to find a job. In these forums, they have developed an internal mechanism for finding jobs. YJ, a middle-aged patient with osteogenesis imperfecta, got her first job through the forum.

*“After I got a computer and learned to type, I searched online for people with disabilities and knew about the Disability Forum, where I met many friends and learned that people with disabilities could find jobs through the Internet. Around 2002, I was given my first job in life, that is, recording beauty information, through a disabled friend’s introduction in the forum.”*

(YJ, a physically challenged person)

In line with the growth of the Internet, the early online employment practices of the people with disabilities were relatively easy, with the majority of jobs confined to text and information entry. Later, with the enrichment of Internet applications, the number of jobs they could do online also increased correspondingly, and diversified forms of jobs such as online “water army,” online writers, retouchers and online store owners gained popularity within the disabled community. Some people with disabilities even said that they had already opened a store through “ebay” to earn money before the popularity of Taobao in 2003.

*“I first sold things online before the rise of Taobao, and I used the platform ebay. I think it was around 2003, shipping with China Post, and there were no other courier companies at that time.”*

(SYX, a physically challenged person)

In 2009, China’s Sina Network launched Sina Weibo, a social networking site with over 100 million registered users in just 2 years, quickly becoming an essential platform for companies and brands to market their products. In this context, people who used to post and be active in forums and portals switched to Weibo marketing accounts, joining their teams to create Weibo accounts with different themes and posting content on time to attract followers. YJ, a person with physical disabilities, said when recalling his experience of running the marketing accounts of Sina Weibo:

*“The operation and management of the marketing number were team-based. At that time, the marketing accounts we made involved several themes, including horoscopes, parenting, interesting stories and anecdotes and so on. To attract followers, we would upload several new posts every day. When the number of followers reached a certain size, we could take advertising to make money.”*

At the same time, the rapid prevalence of Taobao has given rise to many ways of earning a living, such as web-swiping (that is buying products from specific Taobao stores and writing positive consumer reviews for them) and online store customer service jobs. LGT, an impaired person who suffered a spinal cord injury after an accidental fall while playing with a bar during college, served as the store manager of Southeast Asia travel service store on Taobao. During his tenure as store manager, he received an average monthly salary of about 6,000 yuan:

*“Even if I was paralyzed, I still had to live. To reassure my family, I found a job on ‘Tao Job, an Alibaba’s e-commerce job site’ as a travel-specific sales customer service. At that time, I was responsible for the China-Malaysia route planning, I would help customers plan their itineraries, charter buses and book hotels according to their travel time and plans. Later, due to my better performance, I became the store manager in charge of the whole area of Southeast Asia's travel routes.”*

In recent years, the rapid rise of short videos and live webcasts has made live streaming a new mode of online sales, and many people with physical disabilities interviewed have joined the campaign. FQ, a physically challenged person, fond of singing, has been the agent of a professional microphone since July 2019. To increase sales, she insisted on recording short videos of singing with this microphone every day at various landmarks in Wuhan to attract fans. After the number of followers of her short video account exceeded 1,000, she interacted with her fans through live streaming every night from 7 to 11 pm, explaining the functions and features of the microphone to them to facilitate purchase actions.

It is obvious that with the integration and optimization of Internet technology, people with disabilities have broken through the dilemma of narrowing the traditional way of earning a living, and they have ridden on the trend of the development of the Internet to make an innovative way of earning a living and gain practical benefits. It can be said that in the development of the Internet, disabilities are not only passive recipients and beneficiaries, but also participants and creators of the history of the Internet.

### 4.3. “Settling down” and self-worth manifestation

This section attempts to explain how digitalized livelihoods are uniquely meaningful to the people with disabilities. In the Chinese cultural context, the true marker of adulthood is not the age of 18, but rather the age of work, leaving the family of origin, falling in love and getting married, or what the Chinese call “settling down.” However, it is difficult for people with disabilities to enter this stage of life, as most of them experience years of stay-at-home, social isolation, and dependence on their parents for financial support. But this brings a sense of low self-worth and identity, and the vast majority of people with disabilities use the words “low self-esteem,” “feeling like a loser” and “being a drag on their families” to describe their early inability to earn money.

Since the Reform and Opening in 1978, The Chinese Party-state employs neoliberal discourse to encourage individualized citizens ‘to be self-responsible, self-enterprising, and self-governing subjects’, setting up the “labor is the only source of value creation” and “labor is the honorable duty of every citizen who is capable of working (Constitution of the People’s Republic of China).”In this framework, work or labor has become a meaningful way to express the value of the adult individual. Because of their inability to adapt to previous forms of labor, people with disabilities have long been viewed as useless people, a perception that not only affects the general public, but also profoundly imprisons themselves. The advent of Internet technology has provided opportunities to work for people with disabilities, and they are changing the perceptions of their families and themselves about their disabled bodies through digital livelihoods.

YX, a physically challenged person, was paraplegic in 2011 due to a fall. Neither of his parents had a stable job, and the high cost of surgery and rehabilitation after the injury-depleted the family’s savings while also owing more than RMB 100,000 in arrears. To pay off the debts as soon as possible, YX began to look for job through the Internet after being discharged from the hospital. He has done the work of network swiping, part-time training, game coaching, and so on. In the process of game coaching, YX met a friend who operated micro-blog marketing accounts, and then joined the operation team. Now the number of followers of his micro-blog account has reached more than 5 million, with an annual advertising revenue of 300,000 RMB:

*“My friend and I set up two marketing accounts, one for emotional topics, involving gender stories with “pornographic” elements, and another for fitness topics. When we didn’t know how to write a story, we would copy and alter other people’s ideas, and we would also solicit stories from netizens through the Internet. With a huge number of followers and a stable source of income, I used the money I earned to pay off my family's debts and buy a house and a car in Wuhan. My injury dragged the family down. Looking for work online aims at earning money quickly and relieving some pressure on parents. After all, as the only son, I have to take responsibility for the family.”*

As seen in YX’s argument, with the influence of traditional Chinese family values, employment is not an individual problem for people with disabilities, but still a problem for the adult individual, who needs to take responsibility for the family (Pearson et al., 2002). By contributing to the income of their families, people with disabilities proved their abilities and strengthened their self-esteem and self-confidence:

*“After earning money, the greatest feeling is self-confidence and self-worth. When I paid off the money I borrowed from relatives and bought a new house, I felt much more confident and no longer felt inferior when I met my relatives and friends. Even later, some relatives asked me to borrow money, which made me feel dignified.”*

These arguments suggest that for people with disabilities, the significance of earning subsistence capital through the Internet lies not only in the employment itself, but also in the self-worth it creates and the self-confidence and self-esteem it enhances. In particular, as independence, autonomy, and competence become essential elements in defining adulthood in contemporary society, work and employment have become even more essential and carry more connotations of social status and prestige, influencing people’s self-perceptions.

### 4.4. Persistence of “precarious livelihoods”

Before the Internet provided work opportunities for people with disabilities, they also pursued alternative livelihoods. Only by identifying and comparing their digital economy practices throughout their lives can the gains and losses of digital livelihoods be fully and comprehensively explored.

In terms of employment status, the digitalized livelihoods of people with disabilities include two types: self-employment and part-time jobs. The online store opening is a typical representative of self-employed labor, while web swiping and marketing account operations belong to the category of part-time work. Both types of digitalized livelihoods fall under the category of informal employment, where there is no informal employment relationship, not protected by labor laws, and not entitled to employee benefits. At the same time, most of the digital part-time work has low technical content and employment threshold, with no room for career development, while self-employment labor has problems such as tremendous mental pressure and long working hours, so some of them have difficulties in persevering:

*“Only swiping on the Taobao platform does not earn too much money. Especially, with the increasingly strong supervision, this behavior is regarded by the Taobao platform as an act of violation, and accidentally the account will be blocked. I feel that I cannot do it anymore.”*

(ZGM, a physically challenged person)

This indicates that most people with disabilities are still in a state of “precarious livelihood.” This is not fundamentally different from the situation before the rise of the Internet.

Education plays a fundamental role in the digital livelihood of people with disabilities in terms of their individual differences. Those earning more in digital livelihoods tend to be more well-educated people. For example, YX, a person with physical disability, earning nearly RMB 300,000 per year through his Weibo marketing account, has a master’s degree. He said in an interview that even though he had difficulty in going out, he made the most of the Internet to learn about popular cultural phenomena nowadays to improve his knowledge structure, and to stay aware of the popular issues of Internet development. Such basic digital media literacy is an important factor in his ability to stand out among a large number of digital practitioners with disabilities.

Of course, the extent to which government welfare policies are utilized is also an important aspect of whether individuals with disabilities can access better digital livelihoods. One person with a disability, ZQ, who owns five Taobao stores and earns more than 200,000 RMB per year, was able to acquire e-commerce skills through a free government-supported entrepreneurship training program, which led to the success of her business.

*“Before opening a Taobao store, I attended a vocational training program held by the Disabled Persons' Federation. During the class, I learned that people with disabilities could open Taobao stores and learned ways to manage the stores and enhance consumers' trust, which helped me a lot in managing Taobao stores afterward. Later, with the recommendation of the Disabled Persons' Federation, I also participated in 'Magic Beans Mama', a public welfare project jointly sponsored by the Red Cross and Alibaba. Since Taobao is an Alibaba-owned company, my Taobao store was well promoted on the Ali platform and drew the attention of many consumers.”*

People with disabilities like those mentioned above who can take advantage of the opportunities in the digital economy wave are a tiny portion of those interviewed. Taken together, the Chinese government’s measures to support the digital livelihoods of the disabled community, like earlier measures to centralize employment, can only address the livelihood needs of a small percentage of the people with disabilities. Most of them do not know, dare, or are even unwilling to devote more energy to digital livelihoods because of their education, physical fitness and dependence on welfare distribution income (that is basic living allowances), leading to the continued “precariousness” of the livelihoods of people with disabilities.

## 5. Digital prosumer labor of people with disabilities

Due to the influence of audience commodity theory, users’ behavior of using the Internet is called “free labor” as it creates data and flows for the Internet giants and platforms. However, this kind of free labor does not necessarily equivalent to exploitation, there may also produce positive emotions such as relaxation, satisfaction, and happiness (Hardt, 2014). Especially for people with physical disabilities, and McLuhan’s statement that “the medium is an extension of human beings” is not a metaphor, but a factual description. Cell phones and computers have become the electronic organs of people with disabilities, their main interface to the world and their perception of the world. Meanwhile, the extension of their social circle and the improvement of their life skills brought by technology empowerment have reduced their loneliness and enhanced their well-being, which seems to form a “labor compensation mechanism.”

### 5.1. Platform economy and self-esteem brought by independent livelihood

In recent years, although the Chinese government has vigorously promoted the construction of barrier-free facilities, the phenomenon of steep barrier-free ramps and wheelchair-inaccessible restrooms due to irregular construction design is still widely mentioned by the interviewees, which largely restricts the mobility of people with physical disabilities. In addition, many people with disabilities, especially the severely disabled, mostly live with their families to receive care due to their mobility impairment. Yet, as they grow older, people with disabilities also desire their own private space and freedom from family dependence, so they move away from their parents if available. The Internet is an essential tool for dealing with their life problems when living alone. YJ, an osteogenic patient who left her parents to live alone in 2006, describes herself as a heavy Internet user:

*“I am a heavy Internet user. I can’t move easily, so I do a lot of things in my life through the internet. In addition to using the Internet to pay my utility bills, I also buy all my clothes, shoes, and necessities online.”*

(YJ, a physically challenged person)

In the fieldwork, researchers found that many people with disabilities have a strong desire for independence and autonomy and do not like to rely on others for things they can do. For them, “doing things independently” means self-esteem. In October 2021, researchers visited HWZ, a person with a spinal cord injury who had been injured by hot water and needed a skin graft because of the severity of the burns. During the visit, the author saw that only FF, his wife, was taking care of him in the ward, and FF was also a paraplegic with spinal cord injury. The author looked for an opportunity to ask FF about the reason and how she deals with life matters, and FF explained:

*“The parents from both families would like to help, but I refused. We are married and independent. Although I can't move my legs, I can use my hands. I can use the wheelchair to help my husband wash his face and empty the potty. Other things like eating and shopping can be done by takeaways via the internet, which is not difficult in the Internet age.”*

(FF, a physically challenged person)

This shows that in FF’s life, “free labor,” criticized by many digital labor studies in the development of the platform economy, not only brings convenience from the perspective of the general public, but also increases the possibility of living an independent life, and thus strengthens their self-esteem.

### 5.2. Connectivity with society and reduction of loneliness at home

During the interview, YZY, a person with physical disability who has been paralyzed by polio since the age of six, said, “*I used to think that there were probably only four disabled people in the world - me, Shi Tiesheng, Helen Keller, and Zhang Haidi* (the latter three are famous people with disabilities who often appear in the mass media),” and such a narrative that fully illustrated a low degree of socialization of the people with disabilities in China. Many of the severely physically challenged interviewees rarely went out of their homes to participate in socialization activities due to barriers of the environment, limitations of physical functioning, and individual psychological exclusion.

Before the enactment of the *Law on the Protection of Persons with Disabilities* in 1990, some of them were unable to secure even the most basic socialization activity in their life course, “schooling.” CYR, who has a physical disability due to dwarfism, was discouraged from school on the grounds that she is unsuitable for school.

In the structured exclusion of society, people with disabilities are blocked from acquiring knowledge and socialization, and the Internet becomes an alternative channel for them to learn and keep pace with the modernization process of society. They learn about knowledge in history and culture and how to do well socially through the Internet. Also, some of them explain the function of the Internet by exploring the world and appreciating the world:

*“I used to like hiking, climbing, and adventure activities, but since my spinal cord injury in a car accident, I can't participate in these activities. So I often watch adventure videos on relevant platforms to compensate for the pity that I can't explore the world in person.”*

(ZZQ, a person with spinal cord injury)

Digital media is helpful because it provides a learning platform for people with disabilities, suitable to them as opposed to the learning experience in the offline physical space. The emergence of audio broadcasting in mobile applications represents an excellent example. WT, a paraplegic person, often uses online audio radio to listen to books and gain knowledge to prevent pressure sores.

In addition to knowledge acquisition, social exclusion and accessibility issues also affect social participation and social interaction of people with disabilities. “Staying at home” and “having only family members around” are the actual descriptions of the lives of many people with acquired disabilities at the beginning. In this context, the emergence of social media, especially online communities, has given vulnerable individuals scattered in different areas an opportunity to find peers, allowing them to gather online. Almost all of the interviewees mentioned their experiences of joining online communities for people with disabilities:

*“When I was a child, I seldom came into contact with people with disabilities like me, and I always felt that I was a different kind of person and had particularly low self-esteem. After I got a cell phone in high school, I took the initiative to search for people with disabilities on the Internet, finding many forums and QQ groups. I realized that there were many people like me in the world, somehow less alone.”*

NY (a person with a physical disability caused by trauma in the first grade in primary school)

Similar experiences of impairment and social experiences make them able to interact with others on common topics. The online community has increased the opportunities for interaction between people with disabilities by breaking the traditional limits of communication through time and space. After being unable to integrate into the able-bodied world, individuals with disabilities feel a sense of relaxation, understanding and agreement through interactions with the disabled group. From this perspective, the digital labor of the disabled group is an affective labor to some extent, which has the functions of “emotional management” and the establishment of “networked public intimacy” (Kitzman, 2004). They can openly explain highly private contents about physical management of disabled people in online communities, such as defecation, diapers, sexual function and so on. The ‘public’ outlet provided by social media platforms addresses the need to communicate intense personal reactions as well as the highly intimate, often uncomfortable, deeply personal and almost ‘unspeakable’ experiences outside of close personal and face-to-face networks. More importantly, many people with disabilities have also learned lots of subsistence skills through the online community.

*“The able-bodied people can not fully understand what you are going through. Even it is better for them to listen to you. But in such a group, people here can deeply understand your difficulties. If you have any grievances, everyone will comfort you. People here have a strong empathy when knowing others share about their psychological feelings and experiences, and sometimes even relax a lot because they are not alone in this. In addition, people in the group often post information about employment and experiences in caring for bodies. People nearby often play together, much better than going out alone.”*

(LWT, a person with spinal cord injury)

It can be said that the online community for people with disabilities provides a platform for individual members to interact emotionally, and also shares various information resources about disabilities. It provides a platform to communicate their experience, explore their lifestyles, and relieve their loneliness as marginalized people. The online and offline connections pull out the previously “trapped” individuals with disabilities from their homes and promote their social participation.

### 5.3. Making disability “visible”

As “the abandoned mass” (Bauman, 2013) in the context of globalization and modernization, people with disabilities, especially those with severe mobility impairments, have been placed under the guardianship of the state, society and even their families as useless individuals. When direct contact becomes difficult, people rely on the media for their knowledge and understanding of people with disabilities. However, the mass media often present disability with a condescending “able-bodied perspective,” leading to the fact that disabilities are passively subject to the mass media for propaganda, and their “visibility” is entirely dependent on the media agenda. The development of mobile media, 4G technology and short-form video platforms has provided access to cultural production and a good platform for visual participation and self-voice for people with disabilities.

In the field survey, there were 9 persons with disabilities who often shared their lives on short video platforms. By browsing through their short video accounts and video texts, researchers found that almost each of them mentioned “the disability identification” in their profiles, some of them also detailed the reasons, the year and the degree for their disabilities. At the same time, almost all the short video texts were related to disabled life, in which “daily life with disabilities,” “experiences of overcoming barriers,” and “the life journey of people with disabilities” represented their frequent and important contents. For example, short videos posted by FF and XAQ on the Tik Tok platform included the scenes of their life: sweeping the floor in a wheelchair, carrying a delivery in a wheelchair, and making a bed without moving legs, and how to do encountering steps in a wheelchair and so on. In addition to sharing their lives with the public, they presented unfriendly designs in public areas from their own perspectives. As a whole, these videos with specific themes as the core present the physical space where people with disabilities live and their living practices visually. Despite the inevitable conflict between the narrow field of the screen and the wide field of the real space, it provides a vision for viewers of a space close to the reality of the existence of the impaired through the collage of fragments.

Many video producers interviewed said that their behaviors of releasing videos were not only for self-expression, but also to advocate for the disabled community:

*“There are a lot of people with disabilities on Tik Tok, and many of my followers are people with disabilities. The share of my mentality and life, on the one hand, is to make people aware of this group and know that we can also live wonderful life. On the other hand, the purpose is to encourage more people with disabilities to take a peaceful attitude and live a good life through themselves.”*

(XZM, a person with spinal cord injury)

Of course, people with disabilities also cater to the flow logic of the platform when producing videos, adjusting their content design according to the comments and “likes” of users in order to get more attention. But perhaps giving a voice to the disabled and making the disabled visible is a more important issue for the disabled group in the context of Chinese society than the “exploitation” focused on by the existing digital labor.

## 6. Conclusion

The rapid expansion of the platform economy and the digital economy in China has not only transformed the way people work, but it has also absorbed existing disabled groups–who were already underrepresented in the labor market–into the online society’s labor system. This is a sign that Chinese society is continuing to become more digital. The current research on digital labor in China, however, has largely concentrated on concerns of capitalist exploitation and labor control, failing to explain the digital labor practices of marginal groups. It also comprises two entirely separate research frameworks of empowerment and exploitation. This study focuses on the life narratives and experiences of digital labor of people with disabilities, and *via* their self-narratives, examines the significance and impact of digitalized livelihoods and prosumer labor. The research stresses the subjectivity of the labor with people with disabilities as workers, avoids a crude portrayal, and eventually returns to the analytical strategy of critical capitalist exploitation, which offers a fresh viewpoint on digital labor exploitation. Avoid adopting a solitary viewpoint of empowerment or exploitation and instead concentrate on the impact of political, social, cultural, and other numerous aspects on the digital labor of the disabled groups.

This study argues that our understanding of people with disabilities’ engagement in the digital economy and digital consumption should not only focus on the analysis of digital labor patterns and labor processes, but should also be founded on the realities of their existence and pose a further question about why they participate. Due to social marginalization, mobility restrictions brought on by their physical function, and psychological barriers, people with disabilities in China have very low levels of socialization; for some of them, their homes are their only places to reside. The advantages of prosumer labor and digitalized livelihoods in this setting are clear: they improve socialization opportunities, economic rewards, and survival abilities. These advantages have the effect of turning “affective labor” into the dominant paradigm of digital labor for the disabled groups, at least to some extent.

In terms of individual survival skills, with the Internet embedded in all aspects of life, the people with disabilities can handle most of the fundamental problems of daily life with just a mobile phone, which makes it possible for them to live independently. In terms of obtaining livelihood capital, the development of the digital economy has brought many opportunities for “telework,” such as online writers and online store owners. Compared with previous forms of work, digitalized livelihoods are called “suitable work” by people with disabilities, which means that there is no need to enter a “non-inclusive” physical work environment, no need to pay additional costs of clothing, food and transportation. They can finish all their work tasks staying at home. In terms of socialization channels, the Internet’s connectivity and integration functions not only provide a platform for people with disabilities to acquire knowledge, but also enable them to break through the limitations of physical space to connect with the community and society. It can be said that the Internet gives them the opportunities and means to integrate with the social organism, enhancing their sense of value as independent human-beings. Although the research material also shows that the existing digitalized livelihoods are not fundamentally differed from the ‘precarious’ labor conditions in the past, and that the self-presentation of the people with disabilities on social media can easily fall into the flow logic of capital, ‘making disabilities visible’, ‘providing opportunities for people with disabilities to enter society’ and ‘helping them to live with dignity’ are the core values that the digital society brings to the people with disabilities in China.

However, limited by the fieldwork location, this study only explores the digital livelihoods and prosumer labor of urban people with physical disabilities. In the context of the urban–rural imbalance in China’s Internet development, future research could focus on urban–rural differences and differences in the types of disabilities, so as to provide richer material for the digital labor of people with disabilities.

## Data availability statement

The original contributions presented in the study are included in the article/supplementary material, further inquiries can be directed to the corresponding author.

## Ethics statement

The studies involving human participants were reviewed and approved by the Academic Committee of the School of Journalism and Cultural Communication of Zhongnan University of Economics and Law. The patients/participants provided their written informed consent to participate in this study.

## Author contributions

YB has participated in conception and design of the study, the collection, analysis and interpretation of research materials, the drafting of the article, and the critical revision of important intellectual content. XH and RZ participated in the collection and analysis of the research materials. XS has participated in conception and design of the study, the critical revision of important intellectual content. All authors contributed to the article and approved the submitted version.

## Funding

This study was supported by the basic scientific research project of Zhongnan University of Economics and Law (no. 31512210305). Supported by Humanities and Social Sciences of the Ministry of Education Planning Fund (no. 21YJAZH073).

## Conflict of interest

The authors declare that the research was conducted in the absence of any commercial or financial relationships that could be construed as a potential conflict of interest.

## Publisher’s note

All claims expressed in this article are solely those of the authors and do not necessarily represent those of their affiliated organizations, or those of the publisher, the editors and the reviewers. Any product that may be evaluated in this article, or claim that may be made by its manufacturer, is not guaranteed or endorsed by the publisher.
